# The Dichloromethane Fraction of *Sanguisorba tenuifolia* Inhibits Inflammation in Cells Through Modulation of the p38/ERK/MAPK and NF-κB Signaling Pathway

**DOI:** 10.3390/ijms26146732

**Published:** 2025-07-14

**Authors:** Yue Wang, Yiming Lu, Fuao Niu, Siqi Fa, Li Nan, Hyeon Hwa Nam

**Affiliations:** 1 College of Agronomy, Yanbian University, Yanji 133002, China; wangyue9819@163.com (Y.W.); wo4dashuaige2002@163.com (Y.L.); 18043312739@163.com (F.N.); fasiqi123@163.com (S.F.); 2 Department of Crop Life Sciences, College of Agricultural Life Sciences, Jeonbuk National University, 567 Baekje-daero, Deokjin-gu, Jeonju-si 54896, Jeonbuk State, Republic of Korea

**Keywords:** *Sanguisorba tenuifolia*, dichloromethane fraction, anti-inflammatory, MAPK/NF-κB signaling pathway

## Abstract

*Sanguisorba tenuifolia* is a wild plant of the genus *Sanguisorba officinalis*. This study aimed to investigate the regulatory effect of the dichloromethane fraction of *Sanguisorba tenuifolia* on LPS-induced inflammatory responses in RAW264.7 cells, thereby providing a new scientific basis for the medicinal development of *Sanguisorba tenuifolia*. Initially, we used 75% ethanol to crudely extract the roots of *Sanguisorba tenuifolia*, followed by fractional extraction using dichloromethane (CH_2_Cl_2_), ethyl acetate (EtOAc), butanol (BuOH), and distilled water (DW) as solvents. By measuring the inhibitory effects of each fractionated extract on NO production, we determined that the SCE (Dichloromethane fraction of *Sanguisorba tenuifolia*) exhibited the most potent anti-inflammatory activity, leading to its progression to the next experimental stage. Subsequently, we evaluated the effects of SCE on cell viability and LPS-induced inflammatory cytokine secretion in RAW264.7 cells. A rat model of reflux esophagitis was also used to validate the in vivo anti-inflammatory effects of SCE. Additionally, we utilized UPLC/MS-MS to identify and analyze the active components of SCE. The results indicated that SCE could effectively inhibit LPS-induced cellular inflammation by modulating the p38/ERK/MAPK and NF-κB signaling pathways, and also reduced the damage of the esophageal mucosa in rats with reflux esophagitis. UPLC/MS-MS analysis of SCE identified 423 compounds, including 12 active ingredients such as triterpenoids, phenols, and steroids. This discovery not only provides scientific support for the potential of *Sanguisorba tenuifolia* as an anti-inflammatory agent but also lays the groundwork for the development of new therapeutics for the treatment of inflammatory diseases.

## 1. Introduction

Inflammation is a physiological response that occurs when living tissues containing a vascular system are stimulated by various damaging factors. It represents a pathological process characterized by an active defense response. The inflammatory response is one of the body’s immune mechanisms activated in response to damaged tissues or infections, aiming to prevent further harm and block pathogen invasion [1]. During this process, various soluble mediators and inflammatory cells collaborate systematically to eliminate damaging factors. The primary symptoms associated with this basic pathological process include redness, swelling, fever, pain, and dysfunction [2]. By increasing blood flow, enhancing vascular permeability, and attracting immune cells, inflammation facilitates pathogen elimination and promotes wound healing. However, prolonged inflammatory responses can lead to various pathological conditions, such as arthritis, pneumonia, heart disease, and certain cancers [3,4,5]. Therefore, the efficient and rapid treatment of inflammation is a primary objective in the intervention and management of inflammatory diseases.

*Sanguisorba tenuifolia*, a perennial herbaceous plant belonging to the Rosaceae family, is primarily distributed across East Asia, North America, and Western Europe [6]. Its medicinal properties are characterized as slightly cold, with a bitter, sour, and astringent taste, and it is associated with the liver, stomach, and large intestine meridians in traditional Chinese medicine. *Sanguisorba officinalis* L., a species within the same genus as *Sanguisorba tenuifolia*, is widely utilized as a medicinal herb, with its roots being the most commonly employed part. In modern clinical practice, *Sanguisorba officinalis* is frequently used to treat inflammatory conditions such as burns, scalds, proctitis, ulcerative colitis, and nephritis. The roots of *Sanguisorba officinalis* L. are rich in phenols, tannins, triterpenoids, and other active components. Among these, phenolic compounds exhibit antioxidant and anti-inflammatory effects [7], while tannins can promote hemostasis and induce macrophage autophagy to mitigate inflammation [8]. Additionally, triterpenoids are known to regulate immune responses and promote anti-inflammatory phenotypic polarization [9]. Collectively, these constituents exert synergistic pharmacological activities, including anti-inflammatory effects, intestinal mucosal protection, and antioxidant properties. As a member of the same genus as *Sanguisorba officinalis* L., it is plausible that *Sanguisorba tenuifolia* may contain the same active ingredients in its roots, potentially leading to similar pharmacological effects; research on *Sanguisorba tenuifolia* remains incomplete. In this study, LPS-induced RAW264.7 cells were employed to establish an in vitro inflammatory model to assess their anti-inflammatory potential. At the same time, in vivo experiments used a rat reflux esophagitis model to determine its inhibitory effect on inflammation.

This study aims to explore the anti-inflammatory activity of the roots of *Sanguisorba tenuifolia*. The anti-inflammatory effects of its different solvent fractionated extracts were measured, and the optimal anti-inflammatory activity of its dichloromethane fraction was finally determined. Further, it was proved that the dichloromethane fraction regulates the occurrence and development of the inflammatory response by regulating the MAPK/NF-KB signaling pathway. The results of this study provide ideas for innovative drug development, provide basic data for the identification of effective natural medicinal resources, and propose new perspectives and theoretical frameworks for future research.

## 2. Results

### 2.1. Effect of Different Solvent Fractionated Extracts of Sanguisorba tenuifolia on NO Production 

**Figure 1:**
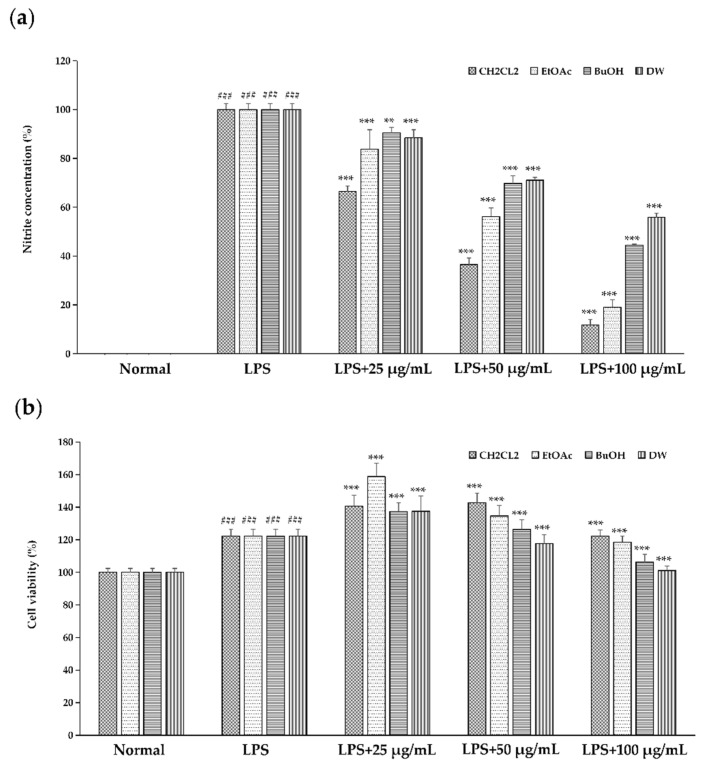
Cells were placed in 96-well plates at a density of 1 × 10^5^ cells/mL. The concentration of the extracts was adjusted to 25 μg/mL, 50 μg/mL, and 100 μg/mL, respectively, and after 1 h of treatment, 1 μg/mL LPS was added and incubated for 18 h. The effect of the graded extracts of Sanguisorba tenuifolia on the amount of NO production for LPS-induced inflammation (**a**) and on the cell viability (**b**). Data are expressed as mean ± standard deviation; LPS was statistically analyzed in comparison to normal cells (^###^ *p* < 0.001), and comparison between each sample and LPS-induced cells (*** *p* < 0.001,** *p* < 0.01).

Different solvent fractionated extracts of *Sanguisorba tenuifolia* demonstrated an inhibitory effect on NO production. The results indicated that LPS induction resulted in a marked increase in NO production, confirming that LPS effectively induced inflammation. Following treatment, NO production was significantly reduced, with extracts from different solvents, including CH_2_Cl_2_, EtOAc, BuOH, and DW, exhibiting varying degrees of inhibition, suggesting that different solvent fractionated extracts of *Sanguisorba tenuifolia* inhibited LPS induction in a dose-dependent manner. The IC_50_ values for the NO inhibition rates of the four solvents were 47.99 μg/mL, 61.63 μg/mL, 89.36 μg/mL, and 108.16 μg/mL, respectively. Among these, the dichloromethane fraction of *Sanguisorba tenuifolia* (SCE) required the lowest concentration for 50% NO inhibition, indicating that SCE exhibited the most potent inhibitory effect (Figure 1a). Importantly, there was no observed impact on cell survival, suggesting the absence of cytotoxic effects (Figure 1b). In summary, SCE was selected as the material for subsequent experiments. 

### 2.2. Effect of SCE on Cell Morphology Changes

**Figure 2:**
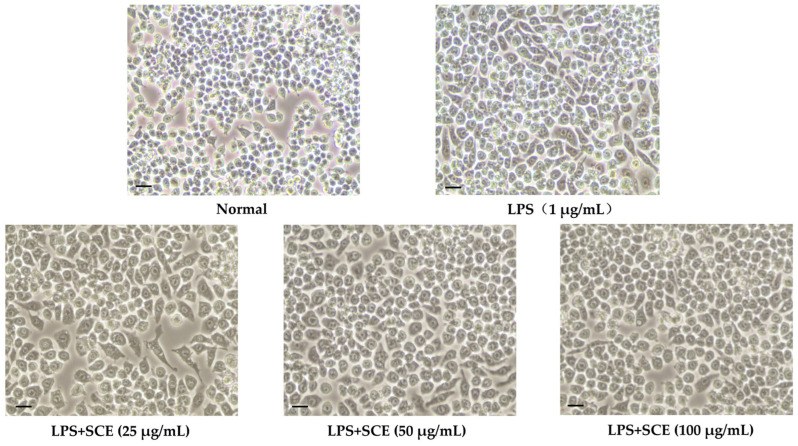
Cells were placed in 96-well plates at a density of 1 × 10^5^ cells/mL. The concentration of extracts was adjusted to 25 μg/mL, 50 μg/mL, and 100 μg/mL, respectively, and after 1 h of treatment, 1 μg/mL LPS was added and incubated for 18 h. The effect of SCE on cell morphology changes.

As illustrated in Figure 2, LPS-induced changes in cell morphology were markedly different from those of normal cells, displaying characteristics such as increased cell spread, volume, irregular shapes, and the presence of pseudopodia. In contrast, SCE treatment significantly mitigated these morphological alterations, and with increasing SCE concentrations, the cell morphology progressively resembled that of normal cells. 

### 2.3. Effect of SCE on the Production of Inflammatory Cytokines and Inflammation-Related Proteins

**Figure 3:**
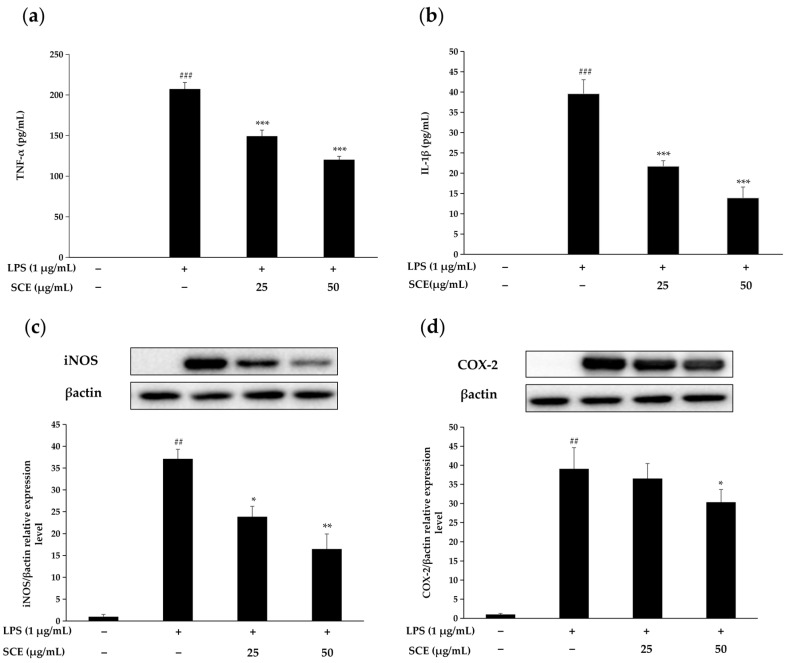
Determination of TNF-α (**a**) and IL-1β (**b**) levels in cell supernatants using ELISA kits. The effects of SCE on the expression levels of iNOS (**c**) and COX-2 (**d**) in LPS-induced RAW264.7 cells were detected by immunoblotting. Data are expressed as mean ± standard deviation, and LPS was statistically analyzed in comparison to normal cells (### *p* < 0.001, ## *p* < 0.01), and comparison between each sample and LPS-induced cells (*** *p* < 0.001, ** *p* < 0.01, * *p* < 0.05).

Figure 3a,b demonstrate that RAW264.7 cells exhibited a notable increase in IL-1β and TNF-α levels following LPS stimulation. In contrast, treatment with 25 μg/mL and 50 μg/mL SCE significantly inhibited the secretion of both IL-1β and TNF-α. Moreover, as the concentration of SCE increased, the degree of inhibition also escalated, suggesting that SCE has a strong inhibitory effect on the production of key inflammatory cytokines.

Figure 3c,d further illustrate that LPS stimulation led to a significant increase in the expression of iNOS and COX-2 proteins in RAW264.7 cells. However, treatment with SCE markedly suppressed the expression of both iNOS and COX-2. Therefore, it can be concluded that SCE effectively mitigates cellular inflammatory responses.

### 2.4. Effect of SCE on Activation of the MAPK Pathway

**Figure 4:**
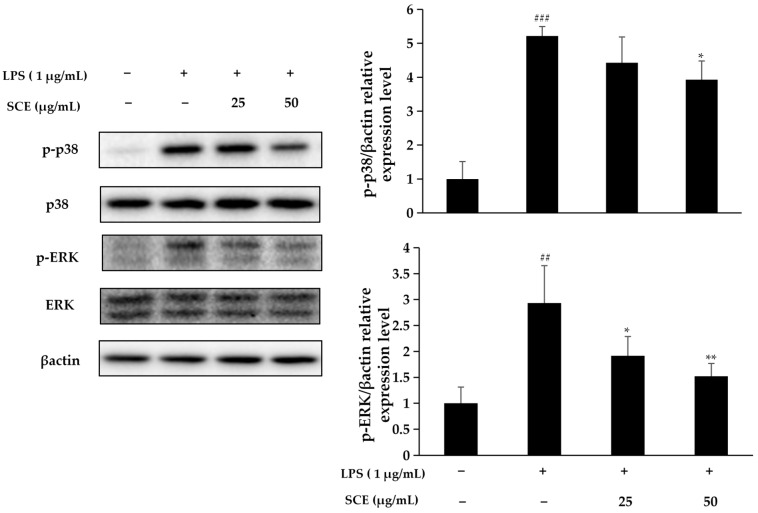
Effect of SCE on p38 and ERK phosphorylation levels in LPS-induced RAW264.7 cells detected by immunoblotting. The data are expressed as mean ± standard deviation. LPS was statistically analyzed in comparison to normal cells (### *p* < 0.001, ## *p* < 0.01), and comparison between each sample and LPS-induced cells (** *p* < 0.01, * *p* < 0.05).

As illustrated in Figure 4, under LPS stimulation, the phosphorylation of p38 and ERK indicates the activation of the p38/ERK/MAPK signaling pathway, which is closely associated with the regulation of inflammatory responses. Notably, SCE treatment significantly reduced the phosphorylation levels of p38/ERK, leading to the inhibition of p38/ERK/MAPK signaling pathway activation. This observation suggests that SCE may possess potential anti-inflammatory effects by specifically targeting this signaling pathway.

### 2.5. Effect of SCE on Activation of NF-κB Pathway

**Figure 5:**
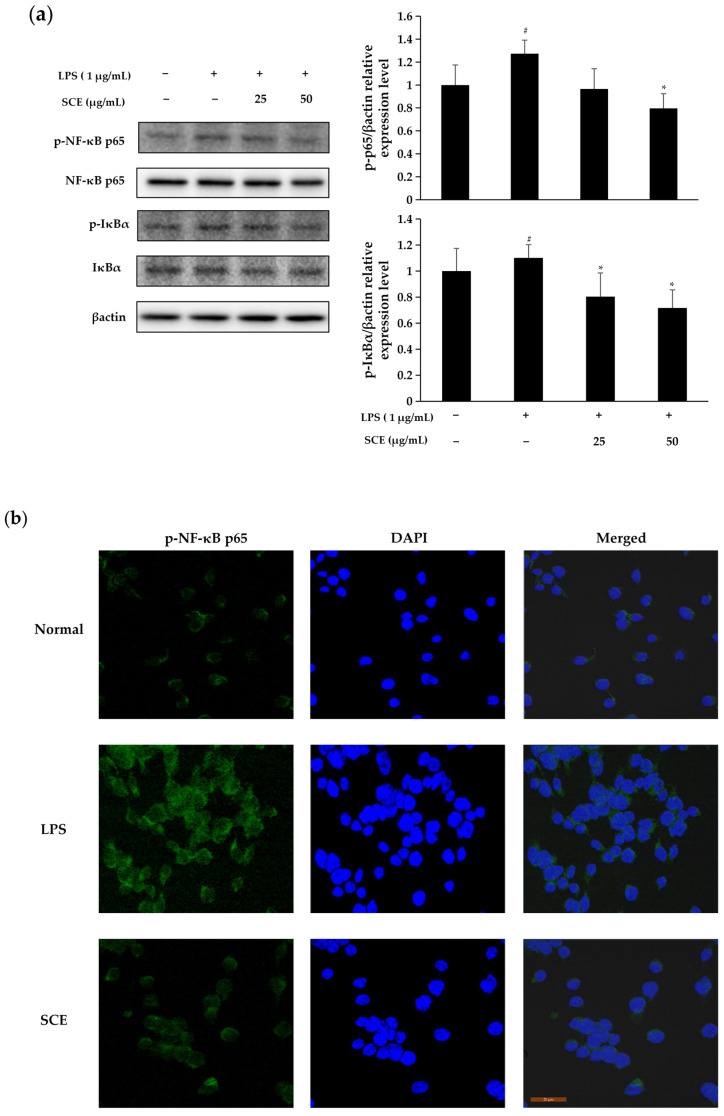
The influence of SCE on the phosphorylation of NF-κB p65 and IκBα (**a**) and the nuclear translocation of (**b**) in RAW264.7 cells stimulated by LPS was examined using immunoblotting and immunofluorescence techniques. Data were expressed as mean ± standard deviation. LPS was statistically analyzed in comparison to normal cells (# *p* < 0.05), and the comparison between each sample and LPS-induced cells (* *p* < 0.05).

According to Figure 5, the nuclear expression of NF-κB p65 increased following LPS stimulation compared to normal cells. Treatment with 50 μg/mL SCE inhibited the nuclear translocation of NF-κB p65 relative to the LPS-stimulated group, indicating that the anti-inflammatory effect of SCE is mediated by preventing NF-κB p65 from entering the cell nucleus. LPS stimulation led to the nuclear translocation of NF-κB p65, accompanied by an increase in IκBα levels and phosphorylation of NF-κB p65. Furthermore, treatment with SCE at a concentration of 1 μg/mL effectively inhibited the phosphorylation of IκBα and NF-κB p65 induced by LPS, ultimately resulting in the suppression of the NF-κB signaling pathway.

### 2.6. Effect of SCE on Esophageal Mucosal Injury in Rats

**Figure 6:**
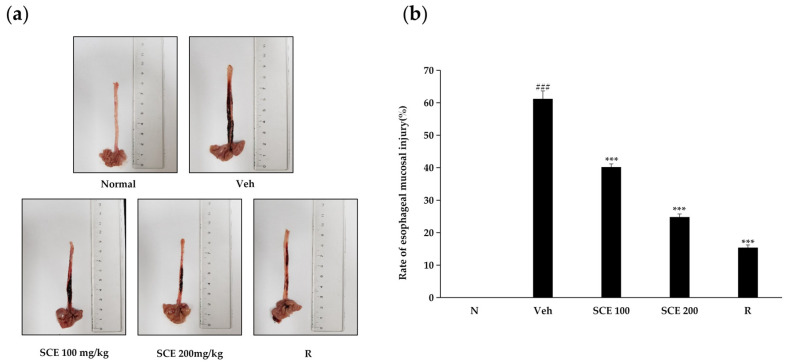
Effects of SCE on esophageal reflux-induced esophageal mucosal damage in rats. Gross (**a**), the ratio of esophageal damage (**b**). N, Normal rats; Veh, RE-controlled rats; SCE 100 mg/kg, RE-controlled rats treated with SCE 100 mg/kg; SCE 200 mg/kg, RE-controlled rats treated with SCE 200 mg/kg and R, RE-controlled rats treated with ranitidine 30 mg/kg. ### *p* < 0.001, *** *p* < 0.001, vs. RE-controlled rats. Data were expressed as mean ± standard deviation.

As illustrated in Figure 6, reflux esophagitis causes significant damage to the esophageal mucosa. Following treatment with two different concentrations of SCE, the inflammatory response was notably inhibited, with greater concentrations yielding more pronounced anti-inflammatory effects. At a concentration of 200 mg/kg, SCE exhibited a level of efficacy comparable to that of the positive control, ranitidine, in mitigating the inflammatory response and the rate of esophageal mucosal injury. These findings suggest that SCE may possess inhibitory effects on reflux esophagitis.

### 2.7. Quantitative Analysis of SCE Extract

**Figure 7:**
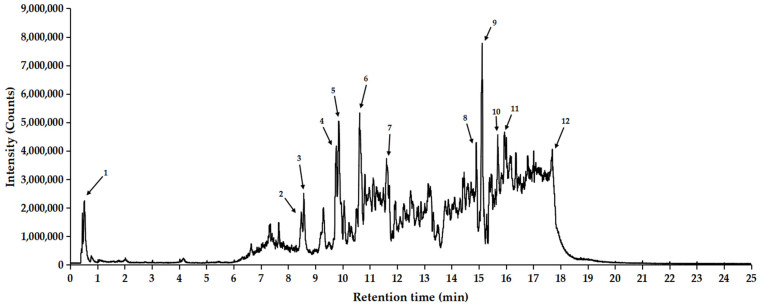
Expanded base peak chromatograms from 0 to 25 min obtained by ultra-performance liquid chromatography-tandem mass spectrometry (UPLC-MS/MS) analysis of SCE.

**Figure 8:**
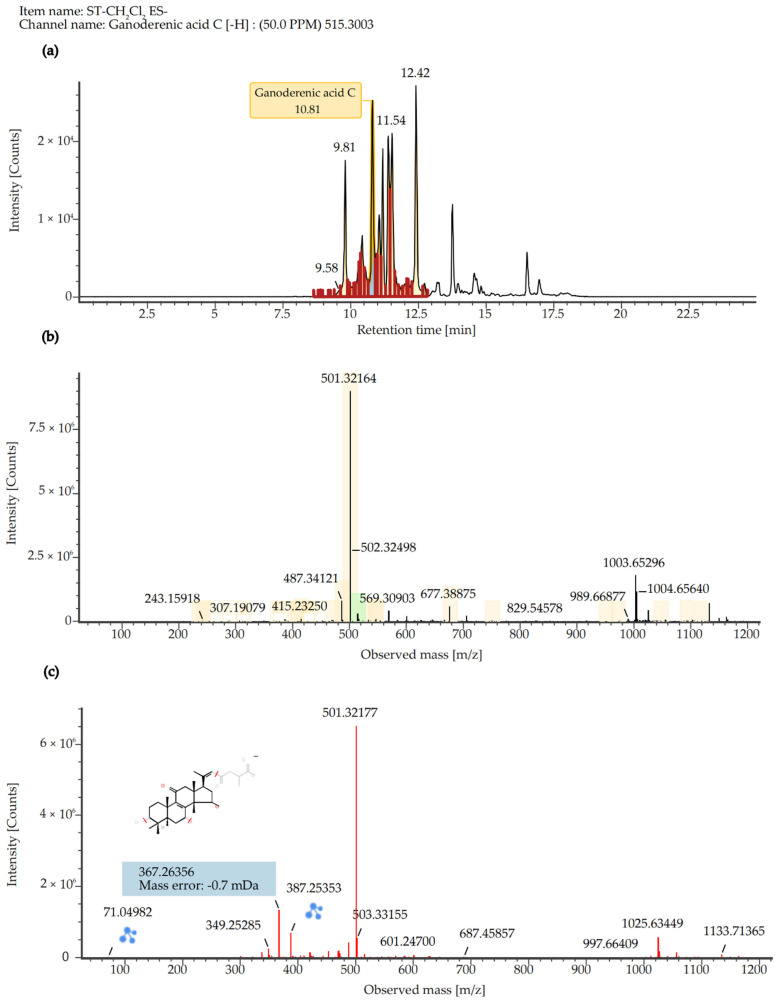
The chromatograph (**a**), molecular ion peak (**b**), and fragment ion peak (**c**) of Ganoderenic acid C in SCE detected by LC-MS/MS (negative ESI source mode) analysis [Color figure can be viewed at wileyonlinelibrary.com].

**Figure 9:**
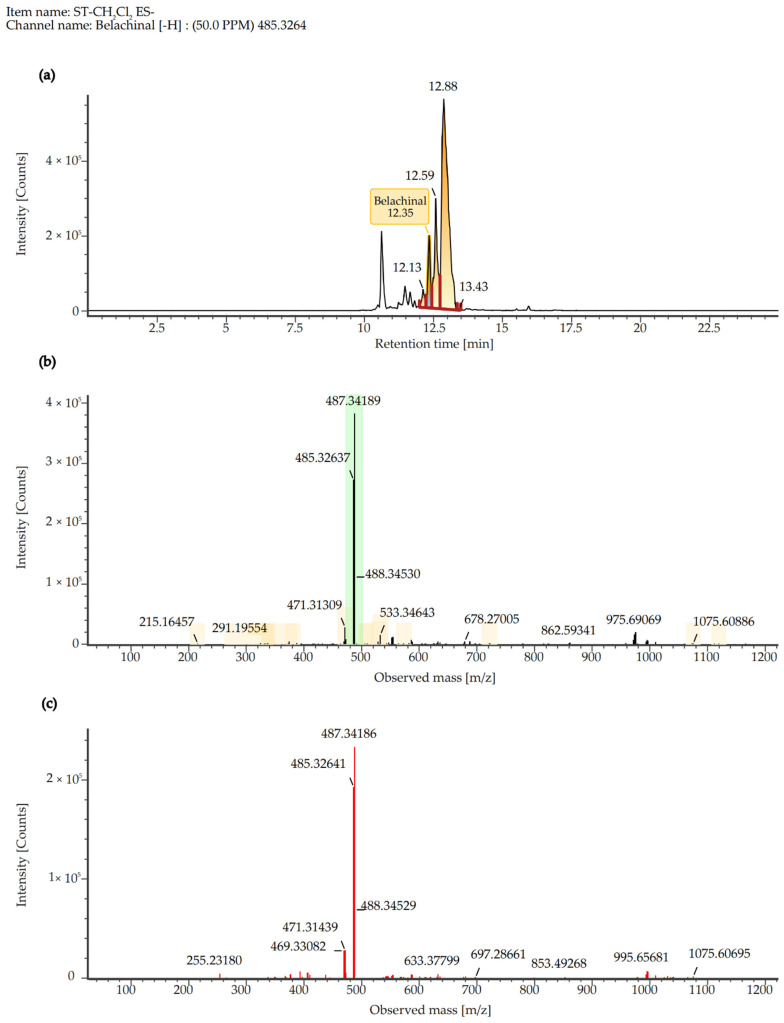
The chromatograph (**a**), molecular ion peak (**b**), and fragment ion peak (**c**) of Belachinal in SCE detected by LC-MS/MS (negative ESI source mode) analysis [Color figure can be viewed at wileyonlinelibrary.com].

To identify the major chemical constituents in SCE, we conducted UPLC-MS/MS analysis. Approximately 12 distinct peaks were observed within a retention time of 0–25 min (Figure 7). The chemical composition, molecular weight, and chemically classified taxa presented in Table 1 correspond to these peaks, which primarily include Tannins (1), Ellagitannins (2), Hydrolyzable tannins (3), Phenols (4), Triterpenoids (5, 6, 8, 10), Pentacyclic triterpene (9), Steroids (7, 11), and Fatty acids (12). Additionally, the active compound Ganoderenic acid C, previously reported in other studies [10,11], was also identified in this study at retention times of 10.43, 11.40, 10.82, and 11.20 min (Figure 8), and its anticancer activity was certified [12]. Similarly, the active compound Belachinal, noted in other research [13], was detected in this study at retention times of 12.33, 10.63, 12.13, 12.59, and 11.83 min (Figure 9); the ingredient is commonly used in anti-inflammatory drugs and has passed the ADMET property as well as ‘Lipinski’s Rule of 5s’ [14]. Tannins, phenols, and triterpenoids are known for their high bioactivity, and their presence may contribute to the significant anti-inflammatory activity of SCE. Furthermore, these compounds may serve as potential natural medicinal materials for the prevention or treatment of reflux esophagitis.

## 3. Discussion

Natural medicines have emerged as a prominent research focus for the potential replacement of traditional anti-inflammatory drugs, attributed to their low toxicity and multi-target characteristics. LPS activates inflammatory signaling pathways, such as NF-κB, by binding to TLRs [15]. This interaction induces the release of mediators, including TNF-α and NO, triggering a systemic inflammatory response that can result in multi-organ failure in severe cases [16,17]. This study investigates the inhibitory mechanisms and active components of SCE in the context of LPS-induced inflammation.

In this study, the LPS-induced RAW 264.7 cell model confirmed that SCE exhibited significant anti-inflammatory activity. Its inhibition of NO production was dose-dependent and markedly superior to that of the other three solvent extracts. Furthermore, SCE effectively reduced the expression of pro-inflammatory cytokines such as IL-1β and TNF-α, as well as iNOS/COX-2 proteins. Notably, SCE did not demonstrate cytotoxicity within the effective concentration range (Figure 1b), suggesting that its anti-inflammatory effects are target-specific. Morphological observations of cells further indicated that SCE could reverse LPS-induced inflammatory phenotypes, such as excessive cell spreading and pseudopodia formation (Figure 2), implying that its protective effect on cellular structures in an inflammatory environment is closely related to functional regulation. Compared to closely related species such as *Sanguisorba officinalis* L., NO inhibitory activity of SCE is comparable to that of ethyl acetate terpene glycosides and ellagitannin, as reported by Su et al. [18]. However, there are notable differences in their solvent classification characteristics. Dichloromethane, a low-polarity solvent, preferentially enriches more fat-soluble pentacyclic triterpenoids and novel ellagitannins. In contrast, traditional polar solvent extracts, such as those from the ethyl acetate phase, primarily contain moderately polar terpene glycosides. The differences in component activity are directly related to chemical polarity: fat-soluble components in SCE more readily bind to the hydrophobic cavity of the iNOS active center due to their hydrophobic structure. Conversely, BuOH or DW exhibit lower activity due to the enrichment of hydrophilic phenolic acids or the degradation of tannins.

NO plays a crucial role as an inflammatory mediator in the inflammatory response, while iNOS serves as a vital enzyme in this process. When cells are stimulated by external factors, they synthesize large amounts of NO, which triggers the inflammatory response; however, excessive NO can have toxic effects on cells [19]. SCE has been found to inhibit NO release by modulating iNOS levels in LPS-induced RAW 264.7 cells. Additionally, TNF-α is one of the cytokines that appears at the initial stage of the inflammatory response [20]. As an early inflammatory mediator, TNF-α induces the upregulation of IL-1β expression. TNF-α contributes to tissue damage by stimulating inflammatory cells and enhancing the production of NO, reactive oxygen species, and other inflammatory compounds [21]. Chen et al. found that elmolin exhibited anti-inflammatory properties in vitro by attenuating the inflammatory mediators NO, TNF-α, and IL-6 [22]. Similarly, terpene glycosides identified by Guo et al. demonstrated comparable anti-inflammatory activity [23]. This study demonstrates that the role of SCE in regulating NO and inflammatory cytokine networks aligns with the aforementioned results. This further substantiates the mechanism of its anti-inflammatory effect by modulating the iNOS/NO pathway and the cascade of inflammatory cytokines.

The MAPK signaling pathway is crucial for cell survival, apoptosis, and the inflammatory response. Within this pathway, there exists a close regulatory relationship between ERK and p38 [24]. Our findings indicated that stimulation with LPS initiated the phosphorylation of both ERK and p38, subsequently activating the MAPK pathway, which plays a pivotal role in regulating inflammation. However, SCE treatment significantly reduced the phosphorylation levels of p38 and ERK, resulting in the inhibition of MAPK signaling pathway activation. This suggests that SCE may disrupt the MAPK signaling pathway by inactivating ERK and p38, thereby inhibiting LPS-induced inflammatory responses. Lee et al. found that the ethanol extract of *Tetracera loureiri* can stimulate the NF-κB and MAPK signaling pathways in macrophages by downregulating LPS, while also inhibiting the expression of pro-inflammatory mediators and cytokines [25]. Wu et al. confirmed that icariin can reduce cell proliferation and inflammation in rheumatoid arthritis by modulating the GAREM1/MAPK signaling pathway [26]. These findings are highly consistent with the regulatory effect of SCE on the MAPK pathway observed in this experiment, collectively supporting the significance of the MAPK signaling pathway as a target for inflammatory intervention. Furthermore, they highlight a common mechanism by which natural products exert anti-inflammatory effects through targeting this pathway.

NF-κB is a crucial signaling factor in the inflammatory response, activated by various extracellular stimuli, including cytokines and products from bacteria or viruses [27]. In its inactive state, NF-κB exists as a dimer in the cytoplasm, bound to IκB. Upon stimulation by LPS, IκBα undergoes phosphorylation and is rapidly degraded, leading to the activation of NF-κB p65. This process facilitates the translocation of NF-κB p65 into the nucleus, where it binds to inflammation-associated genes, initiating the transcription of inflammatory cytokines and thereby promoting inflammation [28]. The results of this experiment demonstrated that LPS stimulation resulted in the nuclear translocation of p65 and concurrently increased the phosphorylation levels of both IκBα and p65. In contrast, treatment with SCE significantly inhibited the LPS-induced phosphorylation of IκBα and p65, as well as the activation of the NF-κB signaling pathway. These findings suggest that SCE may exert anti-inflammatory effects by inhibiting the phosphorylation of IκBα and the nuclear translocation of NF-κB, which aligns with previous research. Yu et al. demonstrated that polyphenolic phytochemicals can alleviate inflammation by targeting the TLR4/NF-κB signaling pathway in cases of intestinal inflammation [29]. In parallel, Yu et al. confirmed that the anti-inflammatory activity of *Sanguisorba officinalis* L. ethanol extract is linked to the inhibition of the NF-κB and AP-1 signaling pathways, as well as the blockade of Src/MAPK activation [30]. This aligns with the mechanism of action of SCE observed in this experiment, thereby reinforcing the central regulatory role of the NF-κB signaling pathway in the anti-inflammatory effects of plant components.

The inflammatory model of rat reflux esophagitis was treated with two different concentrations of SCE, resulting in varying degrees of anti-inflammatory response. The results of the quantitative analysis of SCE (Table 1) were examined and revealed that SCE contains a variety of active ingredients, including polyphenols, terpenes, steroids, as well as tannins, ellagitannins, and hydrolysable tannins, which exhibit high free-radical-scavenging activities, anti-inflammation effects, atherosclerosis prevention, and cancer treatment [31,32,33,34]. The extracts of *Sanguisorba tenuifolia* and *Sanguisorba officinalis* L. share many similar components, indicating that *Sanguisorba tenuifolia* can serve as an equivalent alternative resource for research on related pharmacological activities or clinical applications [35]. 

## 4. Materials and Methods

### 4.1. Materials and Reagents

The plant samples analyzed in this study were collected from Laobai Mountain, located in the Yanbian Korean Autonomous Prefecture of Jilin Province, and were identified as *Sanguisorba tenuifolia* by Quan Xueli, a professor of horticulture at Yanbian University. This experiment employed the mouse monocyte-macrophage cell line RAW264.7 (obtained from ATCC, TIB-71), along with 10% FBS, DMEM culture medium, streptomycin solution, m-IgGk BP-FITC at a dilution of 1:1000 (Santa Cruz Biotechnology, Dallas, TX, USA), DAPI (Sigma-Aldrich, St. Louis, MO, USA), LPS, TNF-α, Solarbio, CCK-8 (DoGenBio Co., Ltd., Seoul, Republic of Korea), a NO assay kit, an ELISA Reader (Multiscan Spectrum, Thermo Scientific, Waltham, MA, USA), IL-1β (R&D Systems, Minneapolis, MN, USA), NF-κB p65, p-NF-κB p65, IκBα, p-IκBα antibody, iNOS antibody, COX-2 antibody, β-actin antibody, and a Leica microscope (Carl Zeiss, Oberkochen, Germany), along with various other materials. 

### 4.2. Sample Preparation

The roots of *Sanguisorba tenuifolia* are dried in a thermostatic oven at 50 °C and then ground into a fine powder to obtain a dry powder sample. A 10 g dry powder sample was extracted, crude extraction was performed with 75% ethanol, followed by fractional extraction, with 100 mL of extraction solve nt at 50 °C for 2 h each time, and repeated 4 times using Dichloromethane (CH_2_Cl_2_), Ethyl acetate (EtOAc), N-butanol (BuOH), and Distilled water (DW) as solvents. The extracts are vacuum-filtered, rotary-evaporated, freeze-dried, and powdered, and then stored at −20 °C until use. Before use, the sample powder was dissolved in DMSO to a concentration of 50 mg/mL, placed in a freezer at −20 °C, and subsequently diluted to the desired concentration for the experiment. 

### 4.3. Cell Culture

After inoculating RAW264.7 cells into DMEM medium with 10% fetal bovine serum and 1% penicillin-streptomycin, they were cultured in a cell culture incubator at 37 °C, 5% CO_2_. Following two regenerations, cells in the logarithmic growth phase were seeded into either 96-well or 6-well plates at varying concentrations for further experimentation. 

### 4.4. Determination of NO Content

Cells inoculated in 96-well plates (cell concentration 1 × 10^5^ cells/mL) were treated with different concentrations of samples, and after 1 h, LPS was added and cultured for 18 h. The supernatant of the finished cell culture solution was collected after centrifugation, and the Griess reagent method was used to determine the nitrite content in the supernatant using NaNO_2_ as a standard to draw a standard curve, and then the content of NO in the solution to be measured was obtained.

### 4.5. Determination of Cell Viability and Observation of Morphological Changes

Cells inoculated in 96-well plates were treated with different concentrations of samples and LPS, and cell viability was determined after 18 h of incubation using the CCK kit according to the instructions. Alterations in cell morphology were documented and imaged using a Leica microscope at 20× magnification.

### 4.6. Determination of TNF-α and IL-1β Content

Cells were seeded into 96-well plates at a concentration of 1 × 10^5^ cells/mL and treated with various samples. After 1 h, LPS was added to the cultures, which were then incubated for 18 h. Following incubation, the cell cultures were centrifuged to collect the supernatants. The supernatants were then diluted and analyzed for TNF-α and IL-1β levels using ELISA kits (R&D Systems, MN, USA), following the manufacturer’s instructions. 

### 4.7. Immunofluorescence Assay

Cells were plated at a density of 2 × 10^5^ cells/well in 6-well plates on a square glass mask. Following pretreatment with 50 μg/mL SCE for 1 h, the cells are then added to 1 μg/mL LPS and receive an additional pretreatment for 30 min. Subsequently, the cells were collected for fixation, permeabilization, and blocking before being incubated with the NF-κB p65 primary antibody (1:200) overnight at 4 °C. This was followed by incubation with the secondary antibody (m-IgGk BP-FITC 1:1000) for 2 h at room temperature. Finally, the cells were stained with DAPI for nuclear visualization, and images were captured using a super-resolution confocal laser scanning microscope at 63× magnification. 

### 4.8. Protein Blotting to Determine Inflammatory Protein Expression

The proteins were extracted from the cells using protein lysate and quantified before being prepared as super samples for protein blotting analysis with a protein quantification kit. Following this, the proteins were separated through gel electrophoresis and transferred onto a PVDF membrane. The membrane was then gently shaken in 5% skim milk for 1.5 h at room temperature. Subsequently, a 1:1000 primary antibody was introduced and left to incubate overnight at 4 °C. The following day, a 1:10,000 secondary antibody was applied and gently allowed to incubate for 2 h at room temperature.

### 4.9. UPLC Mass Spectrometry Analysis and Conditions

The analysis of SCE (Waters, Milford, CT, USA) samples was conducted using ESI combined with UPLC systems. The injection volume was standardized at 5 μL, utilizing an ACQUITY UPLC HSS T3 Column (100 mm × 2.1 mm with a particle size of 1.8 μm). During the separation process, the flow rate was maintained at 0.5 mL/min, and the temperature was controlled at 40 °C. The mobile phase comprised 0.1% formic acid in water (designated as solvent A) and 0.1% formic acid in acetonitrile (designated as solvent B). The components were effectively separated through a precisely controlled gradient elution process: the initial 5 min utilized 97% solvent A, followed by a gradual increase from 3% to 100% solvent B over 5 to 16 min. Subsequently, solvent B was decreased from 100% to 3% between 17 and 19 min, concluding with a return to 97% solvent A over the next 6 min. Sample quality was assessed in full scan mode, covering a scanning range of 50–1200 *m*/*z* with a scan rate of 0.2 s. The capillary voltage was accurately set for both positive and negative ion modes. The capillary voltages in positive and negative ion modes are 2 V and 40 V, 1 V and 40 V, respectively. Leucine enkephalin served as a lock quality criterion, monitored at a flow rate of 10 μL/min. Data processing efficiency and accuracy were ensured using Waters UNIFI V1.71 software.

### 4.10. Rat Reflux Esophagitis Test and Measurement of Esophageal Injury

The experiment was conducted with the approval of the Experimental Animal Welfare and Ethics Committee of the Experimental Animal Center of Yanbian University (Ethics number: YD20240312020). Forty male rats aged seven weeks and weighing 200 ± 20 g were used. The rats lived in a specific environment for one week and were deprived of food for 18 h before surgery and were not allowed to take in water.; they could not help but drink. Rats were randomized into five groups. The settings of each group included a normal group, a vehicle group, an SCE100 mg/kg group, an SCE200 mg/kg group, and a positive control group. Ranitidine (50 mg/kg) was used as a positive control. After the test, the esophageal mucosal injury rate was calculated with the following formula [36]:

Rate of esophageal mucosal injury (%) = [area of esophageal injury (mm^2^)/total area of esophagus (mm^2^)] × 100

### 4.11. Data Processing and Analysis

The experiments were conducted with three replicates independently, and the values are presented as mean ± standard (SD). ANOVA and Fisher’s LSD test were carried out in SPSS 26.0 (IBM Corporation, Chicago, IL, USA). A significance level of *p* < 0.05 was considered.

## 5. Conclusions

The anti-inflammatory properties of SCE were demonstrated in RAW 264.7 cells by attenuating the inflammatory response induced by LPS. It was found that SCE was non-toxic to the cells while effectively reducing the levels of inflammatory mediators and pro-inflammatory cytokines. Furthermore, SCE inhibited the activation of the p38/ERK/MAPK and NF-κB signaling pathways. Notably, SCE exhibited a significant inhibitory effect in the inflammatory model of rat reflux esophagitis. In conclusion, SCE presents a non-toxic and effective natural alternative to mitigate the complications and potential harm associated with conventional anti-inflammatory drugs. These findings strongly supported the exploration of natural medicines as anti-inflammatory therapies and offered new perspectives for the development of safer and more effective therapeutic agents in the future.

## Figures and Tables

**Table 1:** Chemical composition of SCE with expanded base peaks detected by ultra-performance liquid chromatography-tandem mass spectrometry (UPLC-MS/MS) assay.

No.	Component Name	Neutral Mass (Da)	Observe Neutral Mass (Da)	Detector Counts	Adducts	Isotope Match Intensity RMS Percent	Type of Compound
1	2,3-(S)-Hexahydroxydiphenoyl- D-glucose	482.0697	482.0673	6155	-H	960.52	Tannin
2	Corilagin	634.0806	634.0807	4466	+HCOO	48.89	Ellagitannin
3	3-O-Methyl ellagic acid	316.0219	316.0222	139,306	-H	3.53	Hydrolyzable tannins
4	3,3′-Di-O-methylellagic acid	330.0376	330.0377	61,897	-H	4.18	Phenols
5	Ganoderenic acid C	516.3075	515.3003	10,350	-H	33.07	Triterpenoids
6	Belachinal	486.3345	486.3337	95,911	-H	199.14	Triterpenoids
7	Ergosta-4,6,8(14),22- tetraen-3-one	392.3079	392.3058	6764	+HCOO	21.61	Steroids
8	Poricoic acid B	484.3189	484.3185	183,076	-H, +HCOO	365.91	Triterpenoids
9	α-Boswellic acid	456.3603	456.3598	942,668	-H, +HCOO	2.63	Pentacyclic triterpene
10	3β-O-trans-p-Coumaroyl alphitolic acid	618.392	618.3914	190,687	-H	2.79	Triterpenoids
11	Polyporusterone D	460.3189	460.3194	5071	-H	1.82	Steroids
12	α-Hydroxy tetracosanic acid	384.3603	384.3593	34,746	-H, +HCOO	5.07	Fatty acid

## Data Availability

The original contributions presented in this study are included in the article. Further inquiries can be directed to the corre-sponding author(s). Figure 8 and Figure 9’s color figures can be viewed at wileyonlinelibrary.com.

## Abbreviations

ADMET	Absorption, Distribution, Metabolism, Excretion and Toxicity
BuOH	N-butanol
CCK-8	Cell counting kit-8
CH_2_Cl_2_	Dichloromethane
COX-2	Cyclooxygenase-2
DAPI	4′,6′-diamidino-2-phenylindole
DEME	Dulbecco’s modified eagle’s medium
DMSO	Dimethyl sulfoxide
DW	Distilled water
ELISA	Enzyme-linked immunosorbent assay
ESI	Electrospray ionization
EtOAc	Ethyl acetate
FBS	Fetal bovine serum
IL-1β	Interleukin-1β
iNOS	Inducible nitric oxide synthase
LPS	Lipopolysaccharide
NF-κB	Nuclear factor kappa B
NO	Nitric oxide
PVDF	olyvinylidene fluoride
RE	Reflux esophagitis
SCE	Dichloromethane fraction of *Sanguisorba tenuifolia*
SPSS	Statistical product and service solutions
TLRs	Toll-like receptors
TNF-α	Tumor necrosis factor α
UPLC-MS	Ultra-performance liquid chromatography-tandem mass spectrometry
